# High-Flow Nasal Cannula Outside the ICU: A Systematic Review and Meta-Analysis

**DOI:** 10.3390/jcm15010097

**Published:** 2025-12-23

**Authors:** Andrea Boccatonda, Alice Brighenti, Damiano D’Ardes, Luigi Vetrugno

**Affiliations:** 1 Diagnostic and Therapeutic Interventional Ultrasound Unit, IRCCS Azienda Ospedaliero-Universitaria di Bologna, Policlinico Sant’Orsola-Malpighi, via Massarenti n 9, 40138 Bologna, Italy; alice.brighenti3@studio.unibo.it; 2 Institute of “Clinica Medica”, Department of Medicine and Aging Science, “G. D’Annunzio” University of Chieti-Pescara, 66100 Chieti, Italy; 3 Emergency Department, Anesthesia and Intensive Care, Health Integrated Agency of Friuli Centrale, 33028 Tolmezzo, Italy

**Keywords:** HFNC, oxygen, lung, internal medicine, respiratory failure

## Abstract

**Background**: Use of high-flow nasal cannula (HFNC) expanded from ICUs to internal medicine/respiratory wards during and after the COVID-19 pandemic, but safety and effectiveness in non-ICU settings remain uncertain. **Methods**: We performed a systematic review and meta-analysis of adults (≥18 years) initiated on HFNC in non-ICU wards. Primary outcomes were in-hospital (or 28-day) mortality and ICU transfer; where available, we compared mortality for HFNC vs. conventional oxygen therapy (COT) in do-not-intubate (DNI) cohorts. Observational studies and trials were eligible. Random-effects models synthesized proportions and risk ratios; risk of bias (ROBINS-I/RoB 2) and certainty (GRADE) were assessed. **Results**: Ten studies met the inclusion criteria for any-ward HFNC; subsets contributed data to pooled analyses. Across all non-ICU wards (general wards plus step-up IMCU/HDU), pooled mortality was 14.0% (95% CI 4.6–35.5; I^2^ ≈ 92%). Pooled ICU transfer after ward/step-up HFNC start was 20.0% (95% CI 6.3–48.1; I^2^ ≈ 97%). Restricted to internal medicine/respiratory wards, pooled mortality was 19.8% (95% CI 7.1–44.2; I^2^ ≈ 95%) and ICU transfer 31.2% (95% CI 9.9–65.0; I^2^ ≈ 97%). In step-up units (IMCU/HDU), ICU transfer appeared lower and less variable (22.0% [95% CI 16.5–28.8]; I^2^ ≈ 10%), suggesting environment-dependent outcomes. In a multicenter DNI COVID-19 cohort, HFNC vs. COT showed no clear mortality difference (RR ≈ 0.90, 95% CI 0.75–1.08; adjusted OR ≈ 0.72, 95% CI 0.34–1.54). Certainty of evidence for all critical outcomes was very low due to observational design, high inconsistency, and imprecision. **Conclusions**: HFNC outside the ICU is feasible, but it is related to nontrivial mortality and frequent escalation—particularly on general wards—while step-up units demonstrate more reproducible trajectories. Outcomes appear strongly conditioned by care environment, staffing, monitoring, and escalation pathways. Given very low certainty and substantial heterogeneity, institutions should pair ward HFNC with protocolized reassessment and rapid response/ICU outreach, and future research should prospectively compare ward HFNC pathways against optimized COT/NIV using standardized outcomes.

## 1. Introduction

### 1.1. Rationale

High-flow nasal cannula (HFNC) delivers heated, humidified high-flow oxygen with stable FiO_2_ and low-level positive airway pressure [1,2]. During and after the COVID-19 pandemic, the use of high-flow nasal cannula (HFNC) expanded well beyond intensive care units (ICUs), becoming increasingly common in internal medicine and respiratory wards. This shift occurred to preserve ICU capacity and to provide a better-tolerated alternative to conventional oxygen therapy (COT) or non-invasive ventilation (NIV) for patients with acute hypoxemic respiratory failure [3,4,5]. Although several studies have evaluated HFNC in critical care environments, evidence regarding its safety and effectiveness outside the ICU remains fragmented and highly heterogeneous [2].

The existing literature often aggregates patients treated across diverse settings—such as ICUs, emergency departments, intermediate care units (IMCUs), and high-dependency units (HDUs)—making it difficult to isolate outcomes specifically attributable to ward-level HFNC [3,6,7,8,9]. Moreover, the rapid implementation of HFNC during the pandemic occurred in varying organizational models, with inconsistent monitoring capacity, escalation criteria, and patient selection [10,11,12]. As a result, key clinical questions remain unanswered: What is the expected mortality for patients started on HFNC in non-ICU wards? How often do these patients require ICU transfer? And does HFNC offer any advantage over COT in frail do-not-intubate (DNI) patients managed outside the ICU? To address this gap, the present systematic review and meta-analysis focuses specifically on adults initiated on HFNC in non-ICU internal medicine and respiratory wards, separating these settings from step-up units such as IMCUs/HDUs.

This review addresses the following question: Among adults with acute respiratory failure initiated on HFNC outside the ICU, what are the key safety outcomes—specifically mortality and ICU transfer—and, when comparative data are available, how does HFNC compare with conventional oxygen therapy in DNI populations?

### 1.2. Objectives

The primary objective of this systematic review was to evaluate the clinical outcomes of adults with acute respiratory failure who are initiated on HFNC outside the intensive care unit, with a specific focus on internal medicine and respiratory wards. More specifically, this systematic review aims to perform the following:

- Estimate in-hospital (or 28-day) mortality among patients started on HFNC in non-ICU ward settings.
- Determine the proportion of patients requiring ICU transfer after HFNC initiation in internal medicine and respiratory wards.

Mortality and ICU transfer were selected as primary safety outcomes because they represent patient-important endpoints that reflect deterioration requiring escalation of care. Subsequently, we aimed to compare mortality between HFNC and conventional oxygen therapy (COT) in do-not-intubate (DNI) patient cohorts when comparative data were available and to explore differences across care environments, including comparisons between internal medicine/respiratory wards and higher-acuity step-up units (IMCUs/HDUs), to understand how clinical settings influence outcomes.

## 2. Methods

This review follows PRISMA 2020 guidance.

### 2.1. Eligibility Criteria (PICO)

- Population: Adults with acute respiratory failure receiving HFNC outside the ICU.
- Intervention: Ward-initiated HFNC.
- Comparators: COT for DNI comparative data (where available); none for descriptive safety outcomes.
- Outcomes (primary):
  - Primary: Mortality and ICU transfer as safety endpoints.
  - Secondary: Intubation, LOS, physiologic response, comfort, adverse events.
- Study designs: RCTs and nonrandomized studies (prospective/retrospective cohorts, case series ≥10 patients). Exclusions: We excluded ICU-only or ED-only initiation, peri-operative-only cohorts, pediatrics, and IMCUs/HDUs (including surgical). These were included only in pre-specified sensitivity analyses.

### 2.2. Information Sources and Search Strategy

Databases and dates: MEDLINE (Ovid), Embase (Ovid), CENTRAL, CINAHL, Web of Sci-ence/Scopus from inception to 20 September 2025. Full reproducible strategies are listed below for each database, including MeSH/Emtree terms and free text, Boolean operators, and filters: “high flow nasal cannula” OR HFNC OR “high-flow oxygen” OR “high-flow nasal oxygen” OR HFOT) AND (ward* OR “internal medicine” OR respiratory OR “step-up” OR IMCU OR HDU) AND (mortality OR “intensive care” OR intubation* OR transfer OR escalation. The full search strategy is available in Supplementary Data.

### 2.3. Study Selection and Data Extraction

Two reviewers independently screened titles/abstracts and full texts and resolved disagreements by consensus/third reviewer. Data extraction captured study characteristics (setting, period, inclusion criteria), patient demographics/severity, HFNC protocols, comparators, and outcomes. Authors were contacted for missing data when feasible.

### 2.4. Risk of Bias Assessment

Two reviewers assessed RCTs with RoB 2 and observational studies with ROBINS-I across seven domains. Disagreements were resolved by consensus.

### 2.5. Synthesis Methods and Certainty of Evidence

We conducted quantitative analyses separately for single-arm outcomes (mortality and ICU transfer) and for comparative outcomes (HFNC vs. COT in DNI populations). When at least two studies reported an outcome with sufficiently compatible definitions, we performed meta-analysis using random-effects models.

### 2.6. Outcomes and Effect Measures

Single-arm event rates:

For mortality and ICU transfer, we synthesized proportions using random-effects models (DerSimonian–Laird). Proportions were logit-transformed before pooling to stabilize variances, and pooled estimates were back-transformed for interpretation. Studies reporting 28-day mortality were included when in-hospital data were not available, and this was explored in sensitivity analyses.

Comparative analyses:

For the HFNC vs. COT comparison in DNI cohorts, we meta-analyzed risk ratios (RRs) using random-effects models when extractable event counts were available. When studies provided adjusted effect estimates (e.g., odds ratios), these were narratively summarized but not pooled given methodological heterogeneity. When only one comparative study met the inclusion criteria, we reported its results descriptively without pooling.

Heterogeneity assessment:

We quantified between-study heterogeneity using the I2 statistic and τ2. Heterogeneity was interpreted according to standard thresholds and informed whether sensitivity or subgroup analyses were required. Forest plots were visually inspected to assess dispersion and precision across studies. Publication bias and small-study effects were not evaluated, given <10 studies per outcome.

Prespecified sensitivity and subgroup analyses:

We performed sensitivity analyses restricted to the following:

- Internal medicine/respiratory wards only.
- COVID-19-only cohorts.
- Exclusion of step-up units (IMCU/HDU) and surgical contexts.

We also compared pooled estimates between internal medicine/respiratory wards and IMCU/HDU settings to evaluate the influence of clinical environment on outcomes.

Assessment of certainty and risk of bias:

Risk of bias was incorporated qualitatively into interpretation and GRADE certainty-of-evidence ratings for each critical outcome. GRADE domains included risk of bias, inconsistency, indirectness, imprecision, and publication bias (the latter not formally assessed due to limited study numbers).

Software:

All analyses were performed using RevMan (version 5.4.1) with the meta and metafor packages. Numerical results were rounded according to reporting standards for proportional outcomes.

## 3. Results

### 3.1. Study Selection

**Figure 1:**
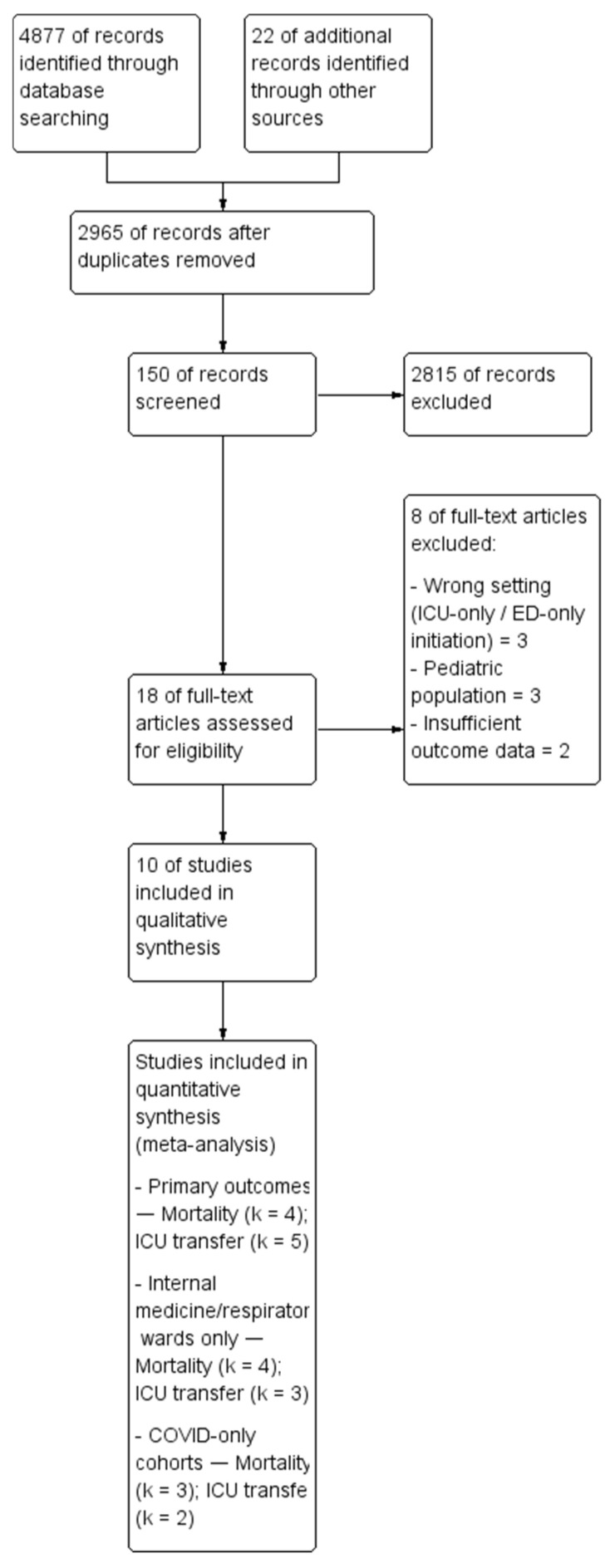
Study flow diagram.

From the records, unique studies were screened; 10 studies were included [13,14,15,16,17,18,19,20,21,22] (Table 1). Of these, three contributed to the internal medicine/respiratory ward mortality meta-analysis, two to the ICU transfer meta-analysis, and one (DNI COVID-19 cohort) permitted between-group comparison. PRISMA flow is illustrated in Figure 1.

### 3.2. Study Characteristics

Studies included single-center and multicenter cohorts from internal medicine/respiratory wards, with variable supervision models (e.g., intensivist-supervised wards, respiratory care units) and COVID-19/non-COVID-19 populations (Table 1). HFNC settings commonly include flows up to 60 L/min and FiO_2_ titration to target SpO_2_. Detailed characteristics appear in Table 1.

### 3.3. Risk of Bias in Included Studies

**Figure 2:**
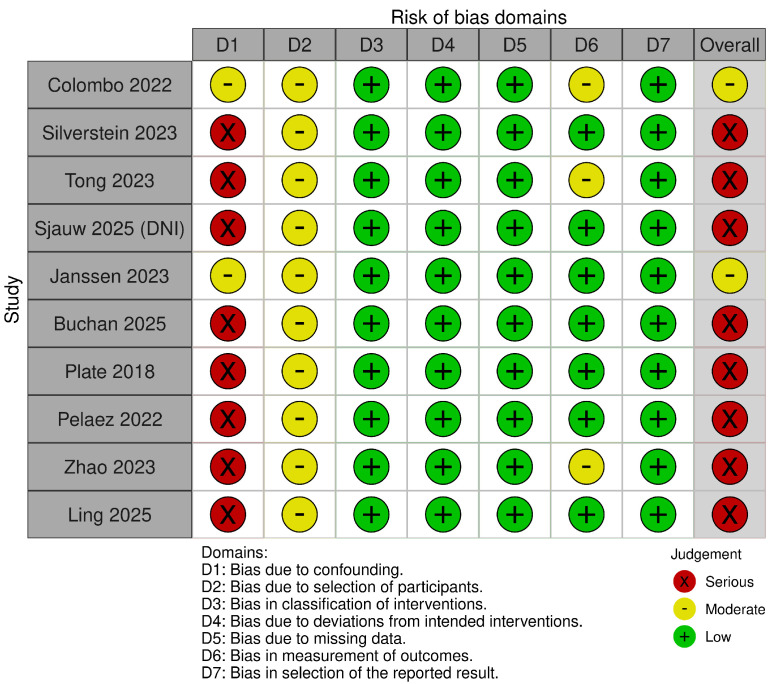
Risk of bias. Traffic light plot [13,14,15,16,17,18,19,20,21,22].

**Figure 3:**
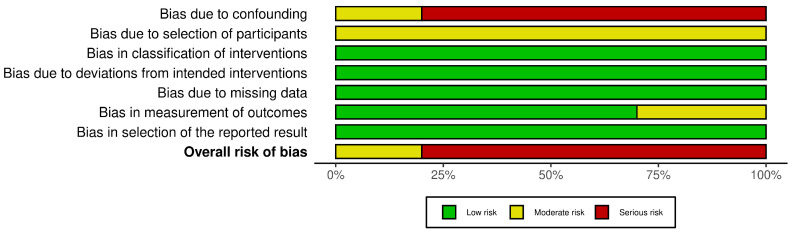
Risk of bias. Summary Plot.

Two reviewers assessed RCTs with RoB-2 and observational studies with ROBINS-I across seven domains; disagreements were resolved by consensus. Most cohorts were at moderate to serious risk of bias (ROBINS-I), chiefly due to confounding by indication, selection bias, and outcome measurement limitations in single-arm designs. Summary assessments are presented in Figure 2 and Figure 3.

### 3.4. Results of Syntheses

**Figure 4:**
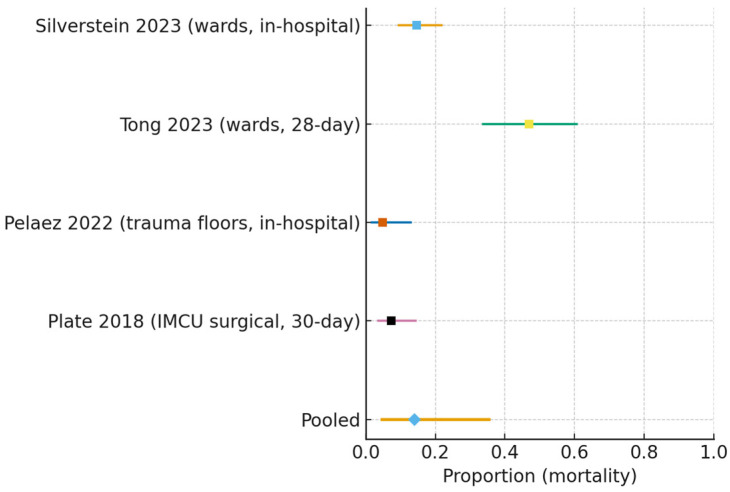
Forest plot of mortality following ward-initiated HFNC (all non-ICU settings) [16,17,19,22]. Individual study estimates (squares) with 95% confidence intervals are shown. The pooled random-effects estimate (diamond) was 0.140 (95% CI 0.046–0.355), with very high heterogeneity (I^2^ = 92.4%, τ^2^ = 1.39).

Mortality (all non-ICU wards):

The pooled mortality was 0.140 (95% CI 0.046–0.355), with high statistical heterogeneity (I^2^ = 92.4%, τ^2^ = 1.39) (Figure 4).

**Figure 5:**
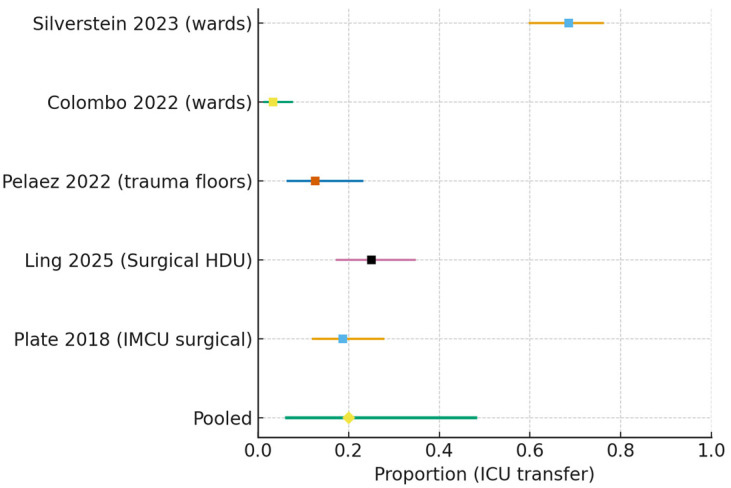
Forest plot of ICU transfer after ward-initiated HFNC (all non-ICU settings) [14,15,16,17,22]. Individual study estimates (squares) with 95% confidence intervals are shown. The pooled random-effects estimate (diamond) was 0.200 (95% CI 0.063–0.481), with very high heterogeneity (I^2^ = 96.7%, τ^2^ = 2.13).

ICU transfer (all non-ICU wards):

The pooled proportion of ICU transfer was 0.200 (95% CI 0.063–0.481), with substantial heterogeneity (I^2^ = 96.7%, τ^2^ = 2.13) (Figure 5).

### 3.5. Subgroup Analyses

#### 3.5.1. Internal Medicine and Respiratory Wards Only

**Figure 6:**
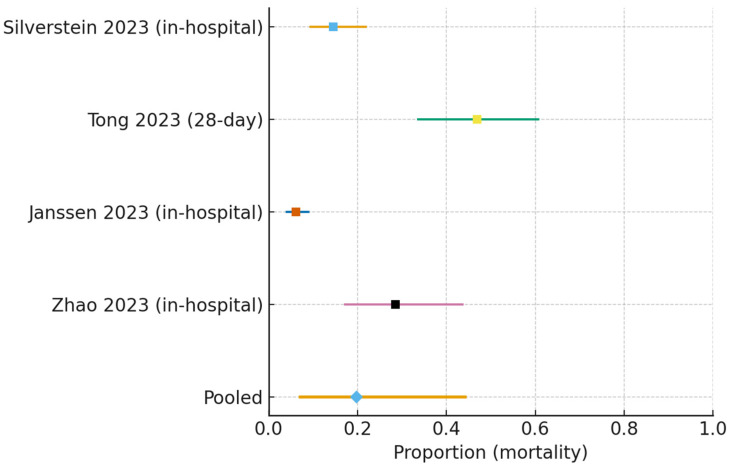
Forest plot of mortality following HFNC initiation on internal medicine/respiratory wards [17,19,20,21]. Study-level estimates (squares) with 95% confidence intervals are presented. The pooled random-effects estimate (diamond) was 0.198 (95% CI 0.071–0.442), based on k = 4 studies. Heterogeneity was very high (I^2^ = 95.0%, τ^2^ = 1.34).

In the internal medicine/respiratory ward subset, pooled mortality was 0.198 (95% CI 0.071–0.442), with high heterogeneity (I^2^ = 95.0%, τ^2^ = 1.34) (Figure 6).

#### 3.5.2. ICU Transfer After Ward-Start HFNC

**Figure 7:**
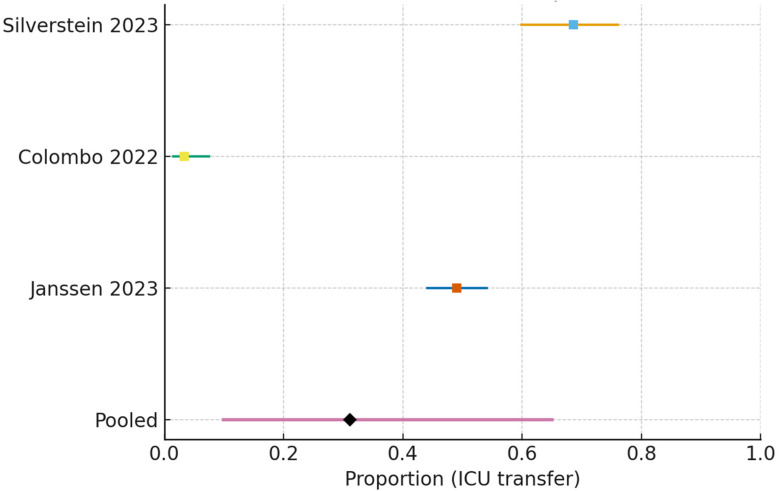
Forest plot of ICU transfer following HFNC initiation on internal medicine/respiratory wards [15,17,21]. Study-level estimates (squares) with 95% confidence intervals are shown. The pooled random-effects estimate (diamond) was 0.312 (95% CI 0.099–0.650), based on k = 3 studies. Heterogeneity was extremely high (I^2^ = 97.4%, τ^2^ = 1.48).

For ICU transfer restricted to internal medicine/respiratory wards, the pooled estimate was 0.312 (95% CI 0.099–0.650) (I^2^ = 97.4%, τ^2^ = 1.48) (Figure 7).

#### 3.5.3. COVID-19-Only Ward Cohorts

**Figure 8:**
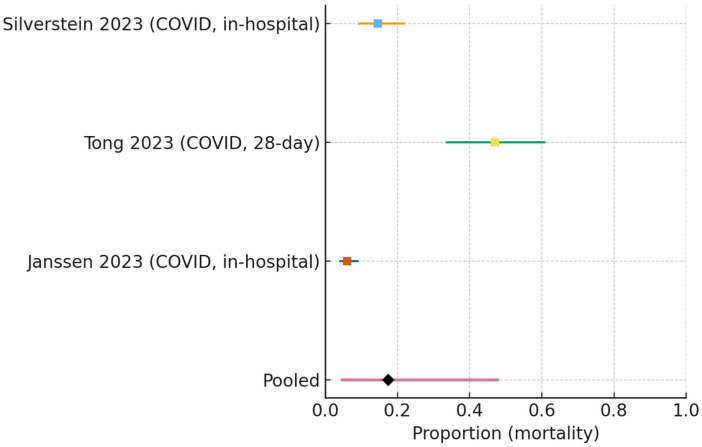
Forest plot of mortality in COVID-19-only cohorts treated with HFNC [17,19,21]. Study-level estimates (squares) with 95% confidence intervals are shown. The pooled random-effects estimate (diamond) was 0.174 (95% CI 0.047–0.478), based on k = 3 studies. Heterogeneity was extremely high (I^2^ = 96.3%, τ^2^ = 1.61), reflecting differences in severity, local thresholds for ICU escalation, and pandemic surge conditions across cohorts.

COVID-19-only ward cohorts yielded a pooled mortality of 0.174 (95% CI 0.047–0.478) (I^2^ = 96.3%, τ^2^ = 1.61) (Figure 8).

**Figure 9:**
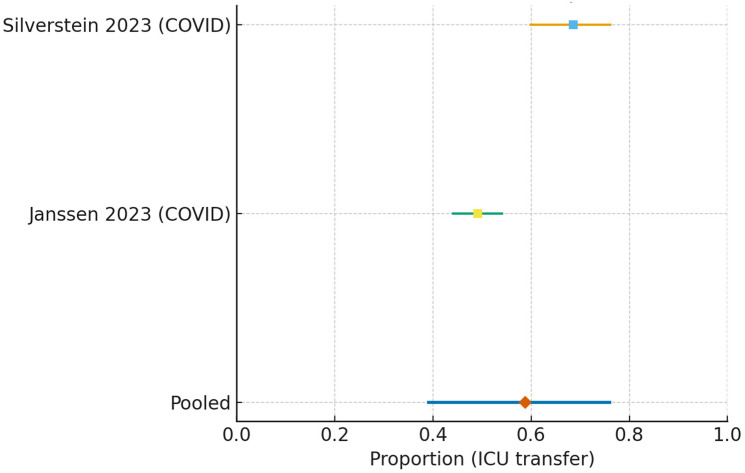
Forest plot of ICU transfer in COVID-19-only cohorts treated with HFNC [17,21]. Study-level estimates (squares) with 95% confidence intervals are shown. The pooled random-effects estimate (diamond) was 0.588 (95% CI 0.391–0.760), based on k = 2 studies. Heterogeneity was high (I^2^ = 92.9%, τ^2^ = 0.31), reflecting differences in patient selection, ward resources, and pandemic surge conditions.

ICU transfer in COVID-19-only cohorts was 0.588 (95% CI 0.391–0.760) with heterogeneity (I^2^ = 92.9%, τ^2^ = 0.31) (Figure 9).

### 3.6. Subgroup Comparison

#### Comparison of IM/Resp Wards vs. IMCU/HDU per ICU Transfer

**Figure 10:**
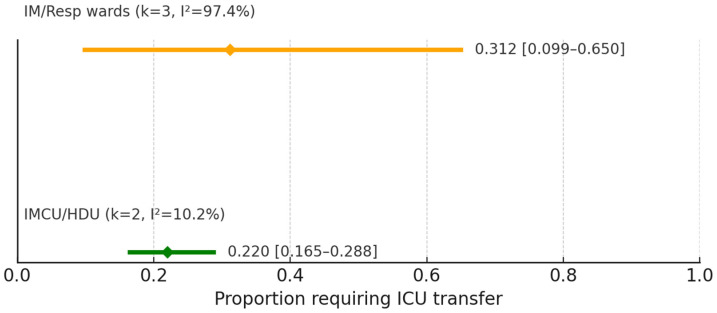
Subgroup comparison of ICU transfer after ward-initiated HFNC (IM/Resp wards vs. IMCU/HDU). Pooled random-effects estimates (diamonds) are shown.

In subgroup analyses, ICU transfer from IMCUs/HDUs had a pooled estimate of 0.220 (95% CI 0.165–0.288) (I^2^ = 10.2%), whereas internal medicine/respiratory wards showed 0.312 (95% CI 0.099–0.650) (I^2^ = 97.4%) (Figure 10).

### 3.7. Certainty of Evidence (GRADE)

#### 3.7.1. Overall Assessment

Certainty-of-evidence assessments using GRADE rated all outcomes as very low, largely due to observational design, inconsistency, and imprecision (Table 2). 

#### 3.7.2. Mortality (All Non-ICU Wards)

Across four observational cohorts, the pooled mortality proportion was 0.140 (95% CI 0.046–0.355), corresponding to 140 deaths per 1000 patients (46–355 per 1000). Certainty of evidence was rated very low due to the following: (i) serious risk of bias (nonrandomized designs with likely residual confounding and selection; several ROBINS-I “serious” judgments); (ii) very serious inconsistency (I^2^ ≈ 92% reflecting differences in epochs, case-mix, and unit configuration); (iii) indirectness, because some cohorts reported 30-day rather than strictly in-hospital mortality and because estimates combined internal medicine/respiratory wards with IMCU/HDU (including surgical units); and (iv) imprecision (wide confidence intervals).

#### 3.7.3. ICU Transfer After Non-ICU Start

Five observational cohorts yielded a pooled ICU transfer proportion of 0.200 (95% CI 0.063–0.481), i.e., 200 per 1000 patients (63–481 per 1000). Certainty was very low, downgraded for serious risk of bias, very serious inconsistency (I^2^ ≈ 97% with markedly different escalation thresholds and surge pressures), and imprecision (very wide CI). Indirectness was judged not serious for this endpoint because it directly reflects ward/IMCU-initiated trajectories.

#### 3.7.4. DNI COVID-19 (HFNC vs. COT)—Mortality

A multicenter comparative cohort study reported an RR of 0.90 (95% CI 0.75–1.08) for death with HFNC vs. COT; the study’s adjusted model suggested an OR of 0.72 (95% CI 0.34–1.54). Certainty was very low given serious risk of bias, indirectness (DNI subgroup, COVID-19-only), and imprecision (confidence intervals compatible with no effect or harm).

#### 3.7.5. GRADE—Summary of Findings (IM/Resp Wards)

In-hospital/28-day mortality (wards):

Across four observational cohorts, the pooled mortality proportion was 0.198 (95% CI 0.071–0.442)—about 198 deaths per 1000 patients (71–442 per 1000). Certainty of evidence was rated very low due to serious risk of bias (nonrandomized designs with likely residual confounding), very high inconsistency across studies, some indirectness (28-day used when in-hospital unavailable), and imprecision (wide confidence intervals) (Table 3).

#### 3.7.6. ICU Transfer After Ward-Start HFNC

Three observational cohorts yielded a pooled ICU transfer proportion of 0.312 (95% CI 0.099–0.650)—about 312 per 1000 patients (99–650 per 1000). Certainty was very low, downgraded for serious risk of bias, very high inconsistency (heterogeneous escalation thresholds and surge phases), and imprecision.

### 3.8. Secondary Outcomes

Secondary outcomes were variably reported and used heterogeneous definitions across studies, precluding consistent pooling. We therefore present a summary table (Table 4). Detailed additional analyses and secondary outcomes are provided in Supplementary Materials.

## 4. Discussion

In this systematic review and meta-analysis of adults started on HFNC outside the ICU, we synthesized outcomes across two complementary scopes: (1) all non-ICU wards (internal medicine/respiratory wards plus ward-managed IMCUs/HDUs, including surgical units where applicable) and (2) a focused subset of IM/Resp wards only. This two-tiered approach helps separate the signal attributable to general medical wards from that of higher-acuity step-up units, where staffing, monitoring, and escalation pathways may differ.

Across all non-ICU wards, pooled mortality was ~14% (95% CI ~5–36%) and ICU transfer ~20% (95% CI ~6–48%). Restricting to IM/Resp wards, pooled mortality was higher, ~20% (95% CI ~7–44%), and ICU transfer ~31% (95% CI ~10–65%). The IM/Resp-only estimates likely reflect resource constraints and lower monitoring intensity compared with IMCU/HDU, whereas the broader non-ICU analysis is diluted by step-up units with greater capability to stabilize patients without ICU transfer. In a DNI COVID-19 subgroup, the single comparative cohort showed no clear survival advantage of HFNC over COT (RR ~0.90, 95% CI 0.75–1.08; adjusted OR ≈ 0.72, 95% CI 0.34–1.54), though modest benefit cannot be excluded.

Heterogeneity was very high for both scopes (I^2^ ≈ 92–97%). Several nonexclusive drivers likely contributed: (i) case-mix (COVID vs. mixed etiologies, age/comorbidity, baseline severity), (ii) unit capability (nurse/RT ratios, continuous monitoring, blood–gas availability), (iii) protocols (HFNC initiation/titration, weaning, criteria and timing for escalation), and (iv) health-system stress (surge periods, ICU bed scarcity), which can shift thresholds for ICU transfer. The setting signal is coherent: when IMCU/HDU cohorts are included, ICU transfer appears lower (≈20%) than in wards-only (≈31%), consistent with enhanced capacity to maintain patients off the ICU. Conversely, mortality appears lower across all non-ICU wards (≈14%) than in wards-only (≈20%), plausibly reflecting earlier escalation within step-up units or different admission thresholds.

The results of this review describe the reported outcomes of HFNC when initiated outside the ICU but do not allow firm conclusions regarding its feasibility or clinical viability in these settings. The substantial heterogeneity, observational design, and very low certainty of evidence limit the degree to which these findings can be generalized or translated into practice recommendations. Rather than indicating effectiveness, the pooled estimates primarily illustrate how outcomes vary widely across different organizational models, monitoring capacities, and escalation pathways. Therefore, the main implication of our findings is not that ward-based HFNC is intrinsically feasible but that its performance appears highly dependent on the care environment. These data emphasize the need to better understand which specific system-level factors (staffing, monitoring, escalation protocols) may influence safety when HFNC is deployed outside the ICU.

Implementation should therefore pair device availability with system design: standardized initiation criteria, early reassessment windows (e.g., 1–2 h checks with ROX trend, respiratory rate, work of breathing), and predefined triggers for NIV/intubation or ICU transfer. Where IMCU/HDU resources are available, shared pathways with ICU outreach teams can reduce delays. For DNI patients, HFNC may align with comfort-oriented goals, but evidence remains at very low certainty and should be framed accordingly.

For both scopes, certainty is very low, driven by serious risk of bias (observational designs, residual confounding), very high inconsistency, and imprecision (wide CIs). For mortality in IM/Resp wards, some indirectness arises where 28-day mortality substituted for strictly in-hospital data. As such, pooled estimates should be interpreted as context-dependent aggregates rather than targets.

Limitations of our analysis are reliance on observational cohorts, variability in outcome definitions (in-hospital vs. 28-day), and incomplete stratification by etiology or gas-exchange phenotype. Small-study effects were not robustly assessable given k.

## 5. Implications for Clinical Practice and Professional Roles

The findings of this review underscore that the effectiveness and safety of HFNC outside the ICU depend not only on the device itself but on the professional practice environment in which it is deployed. For clinicians working in internal medicine and respiratory wards, our results indicate that HFNC cannot be viewed as a standalone intervention; rather, its success hinges on robust monitoring, timely reassessment, and clearly defined escalation pathways. High variability in mortality and ICU transfer rates across studies suggests that staffing ratios, respiratory therapy support, and access to rapid-response or ICU outreach teams play a central role in treatment outcomes.

Moreover, those results reinforce the need for standardized competencies in ward-based management of acute respiratory failure, including familiarity with HFNC titration, early recognition of clinical deterioration, and the use of physiologic response tools (e.g., ROX/SF trends). Interprofessional collaboration—particularly between internal medicine physicians, pulmonologists, intensivists, and respiratory therapists—emerges as essential to optimizing patient trajectories. At the organizational level, hospitals considering or expanding ward-based HFNC programs should ensure that implementation is accompanied by protocolized care pathways, staff training, and clear definitions of failure criteria. Step-up units (IMCU/HDU) demonstrated more consistent outcomes across studies, suggesting that investment in intermediate monitoring capabilities may reduce unplanned ICU transfers and support safer ward-based noninvasive respiratory support.

## 6. Conclusions

HFNC outside the ICU is a possible therapeutic strategy. This review summarizes reported outcomes of HFNC outside the ICU but cannot support definitive statements about feasibility or effectiveness. The available evidence is highly heterogeneous and of very low certainty, underscoring the need for prospective comparative and implementation studies. Outcomes are better contextualized by the care environment: step-up (IMCU/HDU) units appear to mitigate ICU transfer and mortality relative to general wards. Given very low certainty and marked heterogeneity, institutions should adapt these findings to local resources and consider prospective evaluation when scaling non-ICU HFNC programs. Future research should move beyond descriptive single-arm studies and include prospective, comparative designs that evaluate HFNC against optimized conventional oxygen therapy or non-invasive ventilation in non-ICU settings. In parallel, implementation studies are needed to examine how different models of care—such as respiratory wards, internal medicine wards, and IMCUs/HDUs—affect patient outcomes, workflow, and resource use.

In particular, core outcome sets, standardized definitions (e.g., for failure, escalation, physiological response), and harmonized reporting would overcome many of the limitations observed across current studies. Moreover, future work should test protocolized ward HFNC pathways vs. optimized COT/NIV in pragmatic cluster or stepped-wedge trials, with core outcome sets (in-hospital mortality, intubation, ICU transfer, patient comfort) and standardized failure criteria. Comparative evaluations of service models (IM/Resp wards vs. IMCU/HDU; outreach and staffing ratios) are also needed. Beyond COVID-19, dedicated studies in non-COVID-19 AHRF and hypercapnic phenotypes should clarify generalizability.

## Figures and Tables

**Table 1:** Characteristics of included studies. Reported outcomes include mortality (in-hospital or 28-day), ICU transfer, intubation, physiologic/comfort responses, and predictors of failure. Abbreviations: AHRF: acute hypoxemic respiratory failure; APACHE II: Acute Physiology and Chronic Health Evaluation II; CCI: Charlson Comorbidity Index; COT: conventional oxygen therapy; CPAP: continuous positive airway pressure; DNI: do-not-intubate; EIT: electrical impedance tomography; GIM: general internal medicine; HDU: high-dependency unit; HFNC/HFOT: high-flow nasal cannula/oxygen therapy; ICU: intensive care unit; IMCU: intermediate care unit; LOS: length of stay; NIRS: non-invasive respiratory support; NIV: non-invasive ventilation; ns: not significant; PaO_2_/FiO_2_: arterial-oxygen-to-inspired-oxygen ratio; RCU: respiratory care unit; SF ratio: SpO_2_/FiO_2_.

Author/Year	Nation	Setting	Design	Population	Intervention	Comparator	Key Outcomes	Notes
Colombo 2022 [15]	Italy	General wards (nine units, intensivist supervision)	Retrospective observational	150 adults, mild–moderate AHRF (PaO_2_/FiO_2_ 150–300)	HFNC up to 60 L/min, ~FiO_2_ 0.5	None (escalation to NIV/CPAP/ICU if failure)	Failure 19%; ICU transfer 3%; comfort/dyspnea improved; RR decreased	Predictors of failure: higher CCI, higher APACHE II, cardiac failure
Zhao 2023 [20]	Taiwan/China	Respiratory ward	Prospective observational	42 adults with AHRF	HFNC; EIT monitoring	None	Mortality 100% in failures vs. 6% in successes; NLR ≥9 predicts failure; central EIT pattern linked to survival	Small, predictive biomarker focus
Silverstein 2023 [17]	Canada	GIM wards (COVID-19)	Retrospective cohort	124 COVID-19	HFNC on wards	None (capacity analysis)	Mortality 15%; ICU admission 69%; 47% of ICU-admitted were intubated; +20% critical care capacity	Ward HFNC expanded ICU capacity
Janssen 2023 [21]	Netherlands	10 hospitals; ward vs. ICU starters (COVID-19)	Prospective multicenter observational	608 COVID-19 (379 ward, 229 ICU)	HFNC (ward)	ICU initiation	Intubation 53% ward vs. 60% ICU (ns); mortality similar; ward starters had more ICU-free days	Ward HFNC safe; no deaths pre-intubation
Tong 2023 [19]	Hong Kong	Medical wards (COVID-19 Omicron)	Retrospective observational	49 COVID-19	HFNC in ward	None	28-day survival 53%; SF ratio thresholds at 48–72 h predict mortality	Crisis setting; 28-day used
Buchan 2025 [13]	Australia	General wards + RCU (COVID-19)	Cohort study	668 COVID-19 ARF	NIRS (HFNC 36%, CPAP 20%, NIV 2%)	All received COT	Mortality 7.3%; fewer ICU transfers after RCU implementation	Ward/RCU model feasible
Plate 2018 [16]	Netherlands	Standalone surgical IMCU	Case series (retrospective)	96 admissions (mixed; 70% pulmonary)	HFNC in IMCU	None	ICU transfer 18.8%; 30-day mortality 7%; 162 ICU days avoided	Surgical IMCU context
Pelaez 2022 [22]	USA	All hospital floors (trauma)	Comparative vs. historical epoch	63 ≥ 3 rib fractures (22 managed outside ICU)	HFNC available ward-wide	Historical ICU-only (n = 63)	No differences in mortality/LOS; 27% avoided ICU	Trauma focus
Sjauw 2025 [18]	Netherlands	General wards multicenter (DNI COVID)	Multicenter cohort (HFNC vs. COT)	226 COVID-19 DNI (116 HFNC, 110 COT)	HFNC	COT	Mortality 64% HFNC vs. 71% COT; LOS longer in HFNC; adjusted OR ~0.72 (NS)	DNI-specific comparative
Ling 2025 [14]	Singapore	Surgical HDU	Cohort	89 patients (96 HFOT episodes)	HFOT protocolized with staff training	None	Weaning success 67%; ICU-level support 25%	Implementation study

**Table 2:** Certainty of evidence (GRADE) for key outcomes of HFNC initiated outside the ICU. All assessed outcomes—overall mortality, ICU transfer after HFNC initiation, and comparative mortality in DNI COVID-19 patients (HFNC vs. COT)—were graded as very low certainty, reflecting serious risk of bias, high inconsistency, indirectness in some settings, and wide imprecision.

Outcome	Effect (Estimate, 95% CI)	Studies (N)/Design	Certainty of Evidence (GRADE)	Reasons (Downgrading Rationale)
**MORTALITY (ALL NON-ICU WARDS)**	Pooled proportion 0.140 (95% CI 0.046–0.355)	Four studies; observational cohorts	VERY LOW	↓ Risk of bias (ROBINS-I: serious, nonrandomized); ↓ Inconsistency (I^2^ ≈ 92%); ↓ Indirectness (mixed in-hospital/30-day; inclusion of IMCU/HDU, some surgical); ↓ Imprecision (wide CI)
**ICU TRANSFER AFTER NON-ICU START (WARDS/IMCU/HDU)**	Pooled proportion 0.200 (95% CI 0.063–0.481)	Five studies; observational cohorts	VERY LOW	↓ Risk of bias; ↓ Inconsistency (I^2^ ≈ 97%); ↓ Imprecision (very wide CI); Indirectness not serious for this endpoint
**MORTALITY (DNI COVID-19)—HFNC VS. COT**	RR 0.90 (95% CI 0.75–1.08); adj OR ≈ 0.72 (95% CI 0.34–1.54)	One study; observational comparative	VERY LOW	↓ Risk of bias; ↓ Indirectness (DNI subgroup, COVID-19-only); ↓ Imprecision (CI includes no effect and harm)

**Table 3:** Summary of findings (GRADE) for HFNC initiated in internal medicine/respiratory wards. Pooled estimates are derived from observational cohorts and show very low certainty of evidence across all outcomes. In-hospital/28-day mortality was 19.8% (95% CI 7.1–44.2%), and ICU transfer occurred in 31.2% (95% CI 9.9–65.0%). Certainty of evidence was rated very low in all cases due to serious risk of bias, very high inconsistency, indirectness for some outcomes, and wide imprecision.

Outcome	Effect (Estimate, 95% CI)	Studies (N)/Design	Certainty of Evidence (GRADE)	Reasons (Downgrading Rationale)
**IN-HOSPITAL/28-DAY MORTALITY**	Pooled proportion 0.198 (95% CI 0.071–0.442)	Four studies; observational cohorts	VERY LOW	↓ Risk of bias (ROBINS-I: serious); ↓ Inconsistency (I^2^ ≈ 95%); ↓ Indirectness (28-day used when in-hospital unavailable); ↓ Imprecision (wide CI)
**ICU TRANSFER AFTER WARD-START HFNC**	Pooled proportion 0.312 (95% CI 0.099–0.650)	Three studies; observational cohorts	VERY LOW	↓ Risk of bias (ROBINS-I: serious); ↓ Inconsistency (I^2^ ≈ 97%); ↓ Imprecision (very wide CI)

**Table 4:** Summary of secondary outcomes across included studies. HFNC: high-flow nasal cannula; ICU: intensive care unit; COT: conventional oxygen therapy; DNI: do-not-intubate; LOS: length of stay; SF ratio: SpO_2_/FiO_2_ ratio; RR: respiratory rate; EIT: electrical impedance tomography; NLR: neutrophil-to-lymphocyte ratio; PROMs: patient-reported outcome measures.

Outcome	Range or Pooled (If Any)	Notes
**INTUBATION**	~53% ward starters vs. ~60% ICU starters (Janssen 2023 [21], ns); ~47% among ICU-transferred (Silverstein 2023 [17], conditional)	Heterogeneous definitions (overall vs. conditional); composite failure endpoints → no pooling
**HOSPITAL LENGTH OF STAY**	Longer LOS in HFNC vs. COT in DNI cohort (Sjauw 2025 [18]); more ICU-free days for ward starters (Janssen 2023 [21])	Different denominators/time windows; ward vs. hospital LOS inconsistently reported → no pooling
**PHYSIOLOGIC RESPONSE**	SF thresholds at 48–72 h predict mortality (Tong 2023 [19]); RR ↓ and comfort ↑ after HFNC (Colombo 2022 [15]); EIT central pattern and NLR < 9 linked to survival (Zhao 2023 [20])	ROX/SF/EIT metrics not standardized; timings differ; predictive cutoffs vary
**PATIENT COMFORT**	Improved comfort/dyspnea with HFNC (Colombo 2022 [15])	Mostly qualitative; lack of validated PROMs; scales differ
**ADVERSE EVENTS**	Rare/minor; no systematic adjudication	Likely under-reporting; definitions absent

## Data Availability

De-identified data are available on reasonable request.

## Supplementary Materials

The following supporting information can be downloaded at: https://www.mdpi.com/article/10.3390/jcm15010097/s1, reference [23] is cited in the Supplementary Materials (PRISMA 2020 Checklist).

## Abbreviations

HFNC: high-flow nasal cannula; HFOT: high-flow oxygen therapy; IMCU: intermediate care unit; HDU: high-dependency unit; GIM: general internal medicine; NIV: non-invasive ventilation; COT: conventional oxygen therapy; DNI: do-not-intubate; AHRF: acute hypoxemic respiratory failure; LOS: length of stay.
