# Supplementary material for: High-Flow Nasal Cannula Outside the ICU: A Systematic Review and Meta-Analysis
Source: J Clin Med. 2025 Dec 23;15(1):97. doi: 10.3390/jcm15010097 (PMC12786918; doi:10.3390/jcm15010097)
Supplement: Supplementary file 1 [file jcm-15-00097-s001.zip › jcm-3984685-supplementary.pdf]

SUPPLEMENTARY FILES

1.0 SEARCH STRATEGY

MEDLINE (Ovid):

1. exp Oxygen Inhalation Therapy/

2. exp High-Flow Nasal Cannula/

3. (high flow nasal cannula or HFNC or high-flow nasal oxygen or HFNO or high-flow oxygen therapy or HFOT).mp.

4. (heated humidified high flow adj3 oxygen).mp.

5. exp Respiratory Failure/

6. (acute respiratory failure or ARF or AHRF or hypox* or COVID-19 or SARS-CoV-2).mp.

7. (ward* or “general ward*” or “internal medicine” or “respiratory ward*”

or “medical ward*” or non-ICU or “step-up unit”

or IMCU or intermediate care or HDU).mp.

8. (mortality or ICU transfer or escalation or intubation

or noninvasive ventilation or NIV).mp.

9. 1 or 2 or 3 or 4

10. 5 or 6

11. 7

12. 8

13. 9 and 10 and 11

14. 13 not (exp Child/)

Embase (Ovid):

1. high flow nasal cannula/

2. (HFNC or high-flow nasal oxygen or high-flow oxygen therapy

or high-flow nasal cannula or HFOT).ti,ab,kw.

3. heated humidified high flow oxygen.ti,ab.

4. respiratory failure/ or acute respiratory distress syndrome/

5. (acute respiratory failure or AHRF or ARDS or hypox* or COVID-19).ti,ab,kw.

6. (ward* or general ward* or internal medicine or medical ward*
or respiratory ward* or non-ICU or outside ICU or IMCU or HDU).ti,ab,kw.

7. (mortality or ICU transfer or escalation or intubation).ti,ab,kw.

8. 1 or 2 or 3

9. 4 or 5

10. 6

11. 8 and 9 and 10

12. limit 11 to human

CENTRAL:

(high flow nasal cannula OR HFNC OR high-flow nasal oxygen OR HFOT)

AND (respiratory failure OR hypoxemia OR COVID-19)

AND (ward* OR internal medicine OR respiratory ward OR non-ICU)

CINAHL:

("High Flow Nasal Cannula" OR HFNC OR "High-Flow Nasal Oxygen" OR HFOT)

AND ("Respiratory Failure" OR hypox* OR "COVID-19")

AND (ward* OR "internal medicine" OR "medical ward" OR "respiratory ward"
OR non-ICU OR "intermediate care" OR IMCU OR HDU)

Web of Science:

TS = ("high flow nasal cannula" OR HFNC OR "high-flow nasal oxygen" OR HFOT)

AND TS = ("respiratory failure" OR hypox* OR COVID-19)

AND TS = (ward* OR "internal medicine" OR "respiratory ward"

OR non-ICU OR "intermediate care" OR IMCU OR HDU)

Scopus:

TITLE-ABS-KEY ("high flow nasal cannula" OR HFNC OR "high-flow nasal oxygen" OR HFOT)

AND TITLE-ABS-KEY ("respiratory failure" OR hypox* OR COVID-19)

AND TITLE-ABS-KEY (ward* OR "internal medicine" OR "respiratory ward" OR non-ICU

OR "intermediate care" OR IMCU OR HDU)

Grey Literature, Trial Registries & Additional Sources

- ClinicalTrials.gov: search "high flow nasal cannula", "HFNC", "ward", "non-ICU"
- WHO ICTRP
- OpenGrey / ProQuest Dissertations
- Reference lists of all included studies and relevant reviews

Study Filters & Restrictions

Inclusion filters

- Adults ≥18
- HFNC initiated outside the ICU
- Internal medicine/respiratory wards; IMCU/HDU allowed for sensitivity
- Cohort studies, RCTs, prospective, retrospective, case series ≥10 patients

Exclusion filters

- ICU-only initiation
- Emergency department-only cohorts
- Perioperative-only or post-op recovery units
- Pediatrics

2.0 SECONDARY OUTCOMES

Intubation

Across ward-based cohorts, reporting of intubation varied (overall rates vs. conditional on ICU transfer). In the largest multicenter study, Janssen 2023 (21) reported intubation ~53% among ward starters vs. ~60% among ICU starters (non-significant difference). Silverstein 2023 (17) reported that ~47% of patients transferred to ICU were intubated but did not provide an all-comers ward intubation proportion. In Zhao 2023 (20), "HFNC failure" was a composite including NIV and/or intubation and/or death, preventing isolation of a pure intubation rate. Tong 2023 (19), Colombo 2022 (15), Plate 2018 (16), Pelaez 2022 (22), and Ling 2025 (14) did not consistently report ward-level intubation as a standalone endpoint. Given these inconsistencies—and the influence of local escalation thresholds—we did not meta-analyze intubation.

Hospital Length of Stay (LOS)

Reporting was heterogeneous across cohorts. In the DNI COVID-19 comparative study, Sjauw 2025 (18) reported a longer hospital LOS with HFNC vs COT: median 11 [6–18] vs 7 [4–12] days; $p < 0.001$, with the difference remaining after adjustment (direction preserved). In Janssen 2023 (21) (COVID-19, ward vs. ICU starters), unmatched analyses showed more ICU-free days at day 28 for ward starters (median difference ≈ 4 days, $p < 0.001$). Example medians observed included hospital-free days at day 28 around 15 (IQR 1–22) for ward starters vs 12.5 (0–21) for ICU starters, and ICU-free days at day 28 around 24 (13–28) vs. 22 (2–28). However, in matched analyses, hospital-free, ICU-free, and ventilator-free days were similar between ward and ICU starters. Other cohorts either did not report LOS or used incompatible definitions (ward LOS vs. total hospital LOS; inclusion/exclusion of post-ICU days), precluding pooling.

Physiologic Response and Early Predictors

Across studies, physiologic trajectories were emphasized over uniform thresholds, limiting quantitative pooling. In a COVID-19-era respiratory ward cohort, Tong 2023 (19) reported that the SpO_2/FiO_2 (SF) ratio measured at 48–72 h after HFNC initiation discriminated 28-day mortality, with lower SF values independently associated with death; however, cut-offs and timepoints varied within the cohort and were not harmonized with other studies. In a large general-ward series, Colombo 2022 (15) documented early clinical improvement after HFNC, with respiratory rate decreasing from ~25 to ~22 breaths/min within 2–24 h ($p < 0.001$) and patient-reported dyspnea/comfort improving on the Borg/comfort scales ($p < 0.001$). In a physiologic monitoring study on a respiratory ward, Zhao 2023 (20) used electrical impedance tomography (EIT) and found that a predominantly central ventilation pattern during HFNC was associated with better survival, while an NLR < 9 correlated with treatment success; patients who failed HFNC had markedly higher NLR and worse severity profiles (20). ROX index dynamics were reported inconsistently (different measurement windows and FiO_2 titration strategies), precluding standardization across cohorts and preventing meta-analysis of physiologic markers (22).

Patient Comfort

Colombo 2022 (15) directly assessed comfort and dyspnea, reporting improvement after HFNC alongside respiratory rate reduction. Other studies mentioned perceived relief or tolerability qualitatively but lacked standardized scales or pre-specified patient-reported outcome measures.

Adverse Events

Adverse events were rarely reported and generally limited to minor device-related issues (e.g., nasal dryness). No study systematically adjudicated device-related serious adverse events. Under-reporting is likely.
